# Mobile AI-Assisted Oral Lesion Triage for Community Oral Cancer Screening: Prospective Field Evaluation Study

**DOI:** 10.2196/101721

**Published:** 2026-10-20

**Authors:** Hao-Yun Liu, Yu-Cheng Huang, Si-Wei Chen, Ling-Ying Wei, Cheng-Ying Chou, Jang-Jaer Lee, Shyh-Jye Chen, Chien-Chang Lee

**Affiliations:** 1 Department of Medical Imaging, National Taiwan University Hospital Hsin-Chu Branch, Hsin-Chu, Taiwan; 2 College of Medicine, National Taiwan University, Taipei, Taiwan; 3 Department of Medical Imaging, National Taiwan University Hospital, No. 7, Chung-Shan South Road, Taipei, 10002, Taiwan, 886 23123456; 4 Department of Biomechatronics Engineering, National Taiwan University, Taipei, Taiwan; 5 Department of Dentistry, College of Medicine, National Taiwan University, Taipei, Taiwan; 6 Department of Emergency Medicine, National Taiwan University Hospital, Taipei, Taiwan

**Keywords:** artificial intelligence, AI, oral cancer, mobile health, deep learning, diagnostic imaging, telemedicine

## Abstract

**Background:**

AI-based tools for oral cancer screening have shown promising performance in curated image datasets, but their performance in real-world mobile community screening workflows remains less clear.

**Objective:**

This study evaluated the prospective field performance of a parameter-locked, mobile AI-assisted oral lesion triage system embedded in a human-supervised community oral cancer screening workflow.

**Methods:**

We conducted a prospective community-based field evaluation in eastern Taiwan from June 17 to December 31, 2025. Trained nonspecialist personnel acquired standard white-light intraoral images using handheld mobile devices during routine screening. The AI system incorporated on-site image quality assessment and lesion-level risk stratification into green, yellow, and red triage categories. Technically inadequate images after repeated acquisition attempts were classified as ungradable and excluded from performance analysis. Three board-certified oral and maxillofacial specialists established an operational clinical reference standard through structured image review and consensus adjudication. The primary outcome was lesion-level identification of high-risk lesions requiring specialist referral, defined as red versus nonred triage. Diagnostic performance was estimated with Wilson 95% CIs; precision-recall performance and preconsensus specialist agreement were also assessed.

**Results:**

Among 602 screened participants, 4283 interpretable intraoral images were included in expert adjudication and lesion-level analysis. The AI system flagged 68 red lesions; 12 (17.6%) were confirmed as high risk, and 4 additional high-risk lesions were identified during expert review, yielding 16 high-risk events for the primary red-triage analysis. For high-risk lesion identification, sensitivity was 75% (12/16; 95% CI 50.5%-89.8%) and positive predictive value was 17.6% (12/68; 95% CI 10.4%-28.4%); specificity, negative predictive value, and accuracy were 98.6% (3928/3984), 99.9% (3928/3932), and 98.5% (3940/4000), respectively. Area under the receiver operating characteristic curve values were 0.957 for red, 0.931 for yellow, and 0.964 for green triage; precision-recall analysis showed area under the precision-recall curve values of 0.8235, 0.9052, and 0.9476, respectively. Preconsensus specialist agreement was high, with a Fleiss κ of 0.921 and a Gwet AC1 of 0.981.

**Conclusions:**

This mobile AI-assisted oral lesion triage system was feasibly integrated into a real-world community screening workflow operated by trained nonspecialist personnel. In this low-prevalence field setting, red triage showed moderate sensitivity and modest positive predictive value, supporting its use as human-supervised referral-prioritization support rather than autonomous diagnosis. Further multicenter studies with blinded assessment, participant-level outcomes, workflow-efficiency measures, and longitudinal follow-up are needed before clinical impact and scalability can be determined.

## Introduction

Oral cancer (OC) remains a substantial global public health burden and is associated with considerable yet often underrecognized socioeconomic consequences. Major risk factors include tobacco use, excessive alcohol consumption, and betel quid chewing, with a subset of cases associated with human papillomavirus infection [1]. Malignant transformation commonly develops along a continuum of precursor conditions collectively referred to as oral potentially malignant disorders (OPMDs), which encompass clinical and histopathologic alterations of the oral mucosa and carry variable risks of subsequent progression to cancer [2]. Although surgical resection with or without adjuvant therapy remains the cornerstone of treatment, the overall 5-year survival rate for OC has remained approximately 40% to 50% [3]. These observations underscore the importance of early detection and risk-stratified referral, particularly in community screening settings where subtle oral mucosal lesions may be encountered before specialist evaluation.

In Taiwan, a nationwide OC screening program targeting high-risk populations, including individuals with tobacco, alcohol, or betel quid use, has been implemented for more than two decades, reflecting the recognized clinical and public health value of early detection. Nevertheless, the effectiveness of population-level screening remains challenged by the subtle and heterogeneous appearance of early oral mucosal lesions, as well as variability in lesion recognition among frontline screening personnel. These challenges may be amplified in remote community-based screening settings, where access to specialist oral oncology expertise is limited and screening is often performed under field conditions rather than in specialist clinics. Consequently, misclassification or underrecognition of OPMDs remains a long-standing issue [4]. These workflow-level constraints highlight the need for scalable, mobile-compatible tools that can support image acquisition, lesion risk stratification, and referral prioritization while preserving specialist oversight.

With advances in AI and deep learning, image-based tools for detecting oral mucosal lesions have progressed from proof-of-concept classification models toward clinically oriented screening support. Prior studies have shown that AI systems can identify OPMDs and OC from intraoral images with favorable classification or detection performance across diverse datasets [5-13]. Smartphone-based and mobile digital platforms have also been explored as accessible approaches for OC screening in community or resource-limited settings [14,15]. In addition, recent work has incorporated multimodal imaging, lesion segmentation, and risk-based triage frameworks to improve lesion detection, boundary delineation, and referral decision support [16-19]. However, most existing evidence remains focused on model development, internal validation, retrospective datasets, or clinic-based populations, which may not fully capture deployment-related factors such as image acquisition variability, low-prevalence predictive values, and reference standard uncertainty. Prospective evidence on how mobile AI-assisted triage performs when embedded in real-world community screening workflows, particularly with trained nonspecialist image acquisition and on-site quality assurance and expert oversight, remains limited.

To address these clinical and workflow needs, our group previously developed a deep learning system capable of simultaneously identifying multiple coexisting oral mucosal lesions from standard white-light intraoral images collected through Taiwan’s population-based screening program [20]. Unlike approaches centered on single-disease classification, this system was designed to reflect the clinical complexity of routine oral examinations, in which benign lesions, OPMDs, and highly suspicious lesions may coexist within the same individual. In internal validation, the model demonstrated favorable diagnostic performance, supporting further prospective evaluation of its potential role as triage support for community-based screening. However, model performance in curated or development datasets does not necessarily translate into reliable operation in mobile field settings. In community screening workflows, image quality, operator experience, oral cavity exposure, lighting conditions, and lesion prevalence may differ substantially from those observed in controlled datasets. Therefore, prospective evaluation within routine screening workflows is needed to determine whether AI-assisted oral lesion triage can operate reliably under real-world conditions and support referral prioritization without replacing specialist oversight.

Although recent multicenter and clinically oriented studies have reported robust performance of AI systems for OPMD-related detection and classification tasks, much of the existing evidence has been generated from controlled image datasets or clinical populations rather than prospective community screening workflows [21,22]. Direct evidence remains limited regarding how mobile AI-assisted oral lesion triage performs when integrated into real-world field screening, particularly when images are acquired by trained nonspecialist personnel using handheld mobile devices. In this study, we evaluated the prospective field performance of a previously developed, parameter-locked AI-based oral lesion triage system embedded within routine community OC screening. Rather than focusing on model development or diagnostic classification alone, we assessed a human-supervised mobile health workflow that incorporated on-site image quality assurance; green, yellow, and red lesion-level triage; and structured expert adjudication.

## Methods

### Study Design and Setting

This prospective, community-based field evaluation study assessed a previously developed and parameter-locked AI-assisted oral lesion triage system within routine OC screening workflows. The system was deployed from June 17, 2025, to December 31, 2025, during community screening activities in eastern Taiwan, a region with a relatively high OC burden and limited access to specialist oral oncology services. This study was designed to evaluate workflow-integrated performance rather than model development; no retraining, optimization, or post hoc threshold adjustment was performed during the study period. No formal sample size calculation was performed; the sample size reflected all eligible participants screened during the prospective field deployment period. Screening procedures followed the standard operating protocols of Taiwan’s national OC prevention program and were conducted by trained nonspecialist personnel to reflect frontline community practice. Intraoral image acquisition using handheld mobile devices, automated on-site image quality assessment, and AI-generated triage outputs were performed concurrently at screening sites under routine field conditions. Representative images of the community screening environment are provided in Figure S1 in Multimedia Appendix 1.

### Participants and Recruitment

Community residents aged 18 years or older who voluntarily participated in routine OC screening activities were eligible for inclusion. Participants were recruited from community-based screening events rather than specialist referral clinics, thereby reflecting a frontline screening population with a low expected prevalence of high-risk oral lesions. Exclusion criteria were refusal to undergo intraoral imaging, persistently inadequate image quality despite repeated acquisition attempts, or a history of OC treatment resulting in substantial postsurgical anatomic alteration that precluded standardized mucosal assessment. In accordance with Taiwan’s national OC screening protocols, each participant underwent standardized intraoral photographic documentation as part of the screening workflow. Screening-related clinical and imaging data were collected prospectively at the screening sites to reflect real-world community-level heterogeneity. Individual-level demographic variables and behavioral risk factors, including age, sex, tobacco use, alcohol use, and betel quid use, were not collected in the analytic dataset.

### Mobile Image Acquisition and On-Site Quality Assurance

Intraoral images were acquired during routine community screening by trained nonspecialist personnel using handheld mobile devices. Each participant underwent standardized photographic documentation of the oral cavity according to Taiwan’s national screening protocols, with images obtained from predefined mucosal regions to support comprehensive visual assessment (Figure 1). Image acquisition was performed under real-world field conditions without specialized photographic equipment, thereby preserving variability in illumination, patient positioning, oral opening, and operator technique encountered in frontline community screening practice.

**Figure 1.**
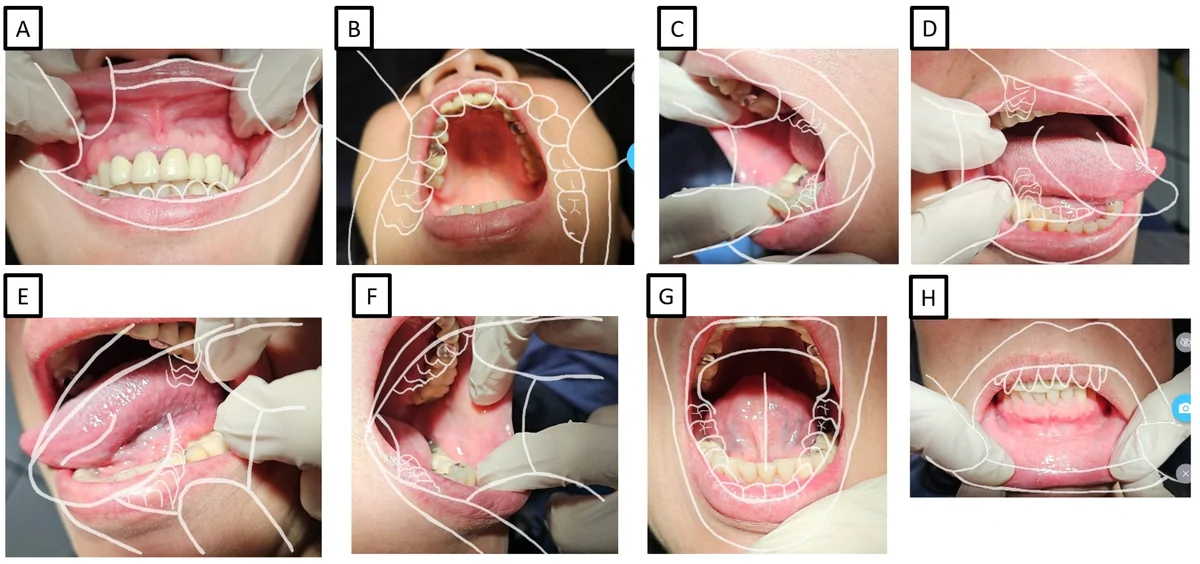
A standard oral photograph sample using augmented reality lines to guide the correct angles and positions for examining different parts of the oral mucosa.

Immediately after acquisition, each image was assessed on-site by the AI-based image quality assurance module. The module evaluated whether the image provided adequate focus sharpness, illumination, mucosal visibility, and absence of motion artifacts for downstream lesion assessment. Images that failed to meet predefined quality criteria triggered immediate repeat acquisition, with up to 3 attempts permitted for each target view. Images that remained inadequate after repeated acquisition attempts were classified as ungradable and were excluded from AI triage output and subsequent performance analysis. This quality assurance step was incorporated into the mobile screening workflow to reduce the impact of technically inadequate images while preserving the practical constraints of community-based image acquisition.

### AI Triage System and Field Deployment Workflow

The AI system evaluated in this study was a previously developed oral lesion triage model trained on 8336 clinical intraoral images collected between 2021 and 2024 at a tertiary medical center. Before field deployment, the model architecture, parameters, and triage thresholds were fixed, and no retraining, fine-tuning, or post hoc threshold adjustment was performed during the study period. The system was designed to identify multiple coexisting oral mucosal lesions within standard white-light intraoral images and generate lesion-level bounding box annotations with risk-based triage outputs to support screening workflow decisions.

For field deployment, the system incorporated 2 sequential modules within the mobile screening workflow. The first was an automated image quality assurance module, which assessed whether acquired images were technically adequate for lesion evaluation and triggered immediate repeat acquisition when predefined quality criteria were not met. The second was a multi-lesion detection and risk stratification module, which classified screening findings into 3 clinically actionable triage categories: green, indicating no actionable lesion or no evident malignant potential; yellow, indicating lesions requiring structured follow-up; and red, indicating lesions requiring specialist referral because of high-risk clinical features. AI-generated outputs were produced on-site as triage support and were not used as autonomous diagnostic decisions. Representative AI-generated outputs for the green, yellow, and red triage categories are provided in Figure S2 in Multimedia Appendix 1. Additional technical details of model development, internal validation, and comparative training outcomes are provided in Table S1 in Multimedia Appendix 1.

### Operational Clinical Reference Standard

The reference standard in this study was defined as an operational clinical reference standard established through structured expert adjudication, reflecting decision-making in community-based OC screening rather than definitive histopathologic diagnosis. Three board-certified oral and maxillofacial specialists independently reviewed the complete intraoral image set for each participant. Reviewers assessed the appropriateness of the AI-generated triage classification and evaluated the presence, location, and risk category of clinically relevant lesions across the full image set.

Reviewers were aware of the AI-generated triage outputs, including green, yellow, and red classifications, during the evaluation process. This design reflected the workflow-integrated nature of the field evaluation and, therefore, constituted an unblinded operational reference standard rather than a blinded diagnostic validation reference standard. In addition to assessing AI-flagged findings, reviewers were instructed to comprehensively examine all original images to identify clinically significant lesions that may not have been detected by the AI system, thereby incorporating potential false-negative findings into the reference standard. Initial independent assessments were recorded for all cases and were used to calculate preconsensus interrater agreement among the 3 specialists. When all reviewers agreed, the concordant classification was adopted. For cases with disagreement, a formal adjudication meeting was conducted, and the final consensus decision was established as the operational clinical reference standard.

Histopathologic examination remains the definitive standard for confirming oral malignancy and epithelial dysplasia. However, systematic biopsy of all lesions or all screened participants is not feasible or ethically appropriate in population-level community screening. In routine screening practice, referral and follow-up decisions are commonly based on clinical visual assessment by experienced specialists, with biopsy reserved for lesions considered clinically suspicious or requiring diagnostic confirmation. Furthermore, in the context of multifocal and heterogeneous OPMDs, localized biopsy sampling may not fully represent overall lesion risk because of inherent sampling limitations [23]. Because this study evaluated AI-assisted triage within a screening workflow rather than final disease diagnosis, structured expert consensus was used to determine clinically relevant risk categories for performance evaluation. This study was designed and reported in accordance with the STARD (Standards for Reporting of Diagnostic Accuracy Studies) 2015 reporting guidelines, and the completed checklist is provided in Checklist 1.

### Outcome Measures

The primary outcome was the lesion-level performance of the AI system for identifying high-risk oral mucosal lesions requiring specialist referral, corresponding to the red triage category. For the primary analysis, each AI-detected or expert-adjudicated lesion was classified using a red vs nonred framework, and lesion-level 2 × 2 contingency tables were constructed using expert adjudication as the comparator. Given the low expected prevalence of high-risk lesions in community screening, interpretation of the primary red triage outcome emphasized sensitivity and positive predictive value (PPV).

Secondary outcomes included category-specific lesion-level performance for the yellow and green triage categories, image-level agreement between AI outputs and expert adjudication where applicable, and discriminative performance based on AI detection confidence using receiver operating characteristic (ROC) and precision-recall analyses. The 3 triage categories were defined according to clinical actionability and were informed by previously reported gradients of malignant transformation risk in OPMDs [24-28]. In this framework, red indicated lesions requiring specialist referral because of high-risk clinical features; yellow indicated lesions requiring structured follow-up because of low to intermediate risk features; and green indicated no actionable lesion or findings without evident malignant potential.

For category-specific analyses, each triage level was evaluated using a one vs the rest framework, with red compared with nonred categories, yellow compared with nonyellow categories, and green compared with nongreen categories. False-negative cases in the red category were further reviewed descriptively to characterize potential failure patterns relevant to real-world mobile screening deployment.

### Statistical Analysis

All analyses were conducted according to the predefined outcome framework. The primary analysis evaluated lesion-level performance of the AI system for identifying high-risk oral mucosal lesions requiring specialist referral using the expert-adjudicated lesion-level classification as the comparator. Secondary analyses included category-specific lesion-level performance for the yellow and green triage categories, as well as image-level agreement between AI outputs and expert adjudication where applicable.

For each analysis, diagnostic performance metrics, including sensitivity, PPV, specificity, negative predictive value (NPV), and overall accuracy, were calculated with corresponding 95% CIs using the Wilson method. Discriminative performance was further assessed using ROC curves and the area under these curves (ROC-AUC) based on AI detection confidence scores for each triage category. Precision-recall curves and the area under these curves (PR-AUC) were also generated to evaluate positive triage performance in the low-prevalence screening setting. Preconsensus interrater agreement among the 3 specialists was summarized using the complete agreement rate, average pairwise observed agreement, Fleiss κ, and Gwet AC1.

All analyses were descriptive in nature, and no formal hypothesis testing was performed. Given the prospective field evaluation setting and the exploratory objective of workflow-integrated assessment, statistical comparisons between groups were not prespecified. Analyses were conducted using the Python software (version 3.11; Python Software Foundation) with standard scientific computing libraries.

### Ethical Considerations

This study was approved by the Research Ethics Committee of National Taiwan University Hospital (approval number NTUH 202504075 RIND). All procedures were conducted in accordance with local regulations and the principles of the Declaration of Helsinki. Screening-related clinical and imaging data were deidentified before analysis and stored in secure institutional systems. Participation was voluntary, and informed consent was obtained according to the approved study protocol. No financial compensation was provided to participants.

## Results

### Participant and Image Flow

Between June 2025 and December 2025, a total of 602 participants underwent community-based OC screening. In accordance with the standardized screening protocol, multiple intraoral images were acquired for each participant, with a mean of approximately 8 images per participant. Of 4816 acquired intraoral images, 533 (11.1%) were excluded because of acquisition failure or inadequate quality, leaving 4283 (88.9%) interpretable images for expert adjudication and lesion-level performance analysis. The participant and image flow is summarized in Figure 2.

**Figure 2.**
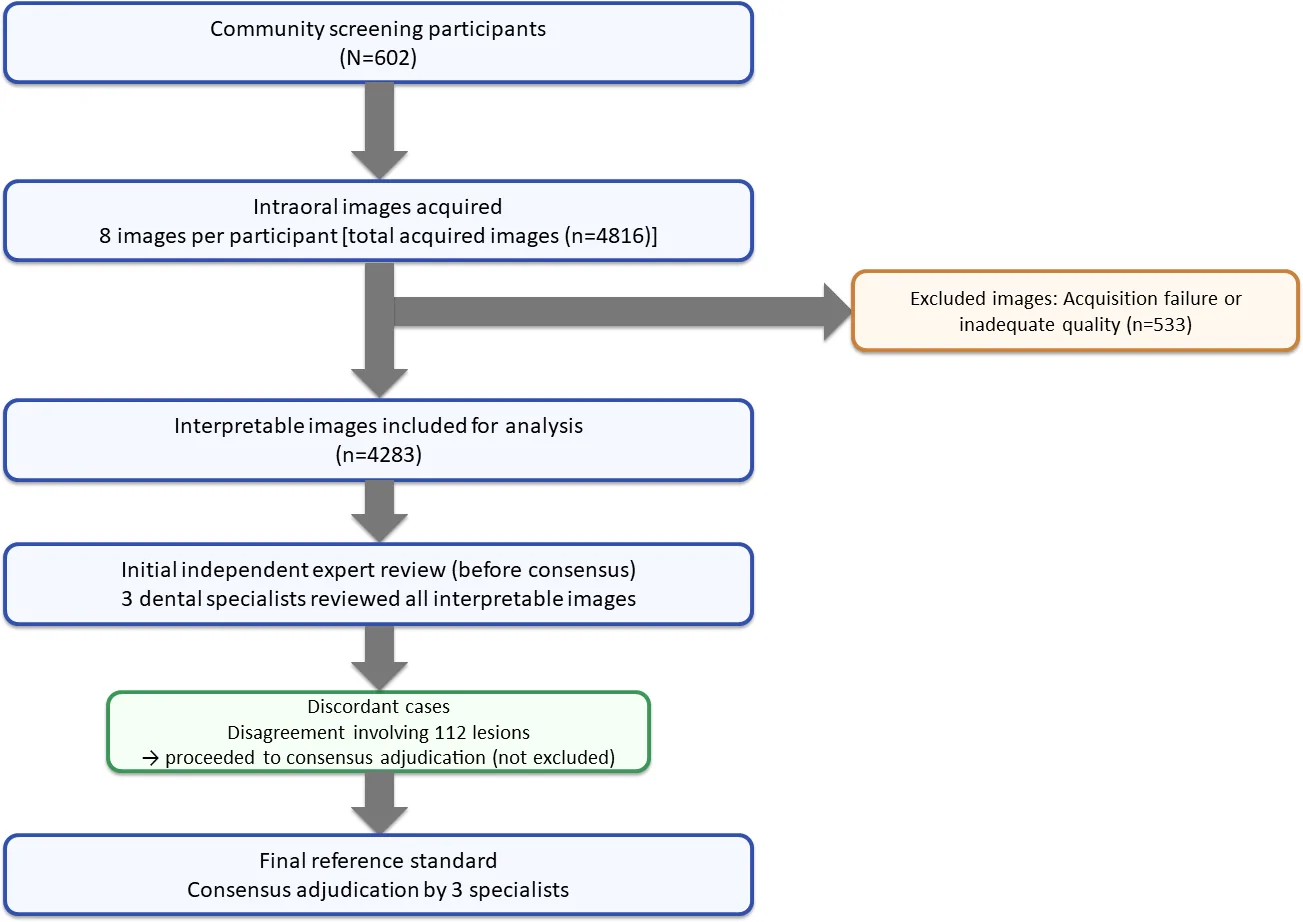
STARD (Standards for Reporting of Diagnostic Accuracy Studies)–consistent participant and image flow diagram.

### Expert Adjudication and Final Analysis Set

All interpretable images were initially reviewed independently by 3 board-certified oral and maxillofacial specialists without discussion. During this initial review, cases for which all 3 specialists reached the same initial classification were summarized as a preconsensus process measure, as shown in Table 1. Preconsensus lesion-level agreement among the 3 specialists was high, with a complete agreement rate of 97.5%, a Fleiss κ of 0.921, and a Gwet AC1 of 0.981. This preconsensus summary was used to describe the initial concordant expert review process and was not used as the primary outcome analysis. Among these concordant initial assessments, 91% (3898/4283) of the images were classified as showing no detectable lesion.

**Table 1.** Lesion-level performance based on concordant initial independent expert assessments before consensus adjudication.

	TPs^a^, n	FPs^b^, n	FNs^c^, n	TNs^d^, n	Sensitivity (%; 95% CI)	Specificity (%; 95% CI)	PPV^e^ (%; 95% CI)	NPV^f^ (%; 95% CI)	Accuracy (%; 95% CI)
Red	10	12	4	3898	71.4 (45.4-88.3)	99.7 (99.5-99.8)	45.5 (26.9-65.3)	99.9 (99.7-100)	99.6 (99.3-99.7)
Yellow	240	16	9	3898	96.4 (94.0-98.1)	99.6 (99.3-99.7)	93.8 (90.1-96.2)	99.8 (99.6-99.9)	99.4 (99.2-99.6)
Green	206	4	16	3898	92.8 (88.6-95.5)	99.9 (99.7-100)	98.1 (95.2-99.3)	99.6 (99.3-99.7)	99.5 (99.3-99.7)

a TP: true positive.

b FP: false positive.

c FN: false negative.

d TN: true negative.

e PPV: positive predictive value.

f NPV: negative predictive value.

Disagreement among the 3 specialists was identified for 112 lesions, indicating that a subset of oral mucosal findings remained clinically equivocal during independent expert assessment. These discordant lesions subsequently underwent formal consensus adjudication to establish the final operational clinical reference standard. After consensus adjudication, all interpretable images, including those with initial discordance, were incorporated into the final lesion-level analysis. In the final adjudicated dataset, 91.7% (3928/4283) of the images were confirmed as not containing detectable lesions, and the final adjudicated performance is shown in Table 2.

**Table 2.** Final adjudicated lesion-level performance after expert consensus.

	TPs^a^, n	FPs^b^, n	FNs^c^, n	TNs^d^, n	Sensitivity (%; 95% CI)	Specificity (%; 95% CI)	PPV^e^ (%; 95% CI)	NPV^f^ (%; 95% CI)	Accuracy (%; 95% CI)
Red	12	56	4	3928	75.0 (50.5-89.8)	98.6 (98.2-98.9)	17.6 (10.4-28.4)	99.9 (99.7-100)	98.5 (98.1-98.8)
Yellow	281	64	22	3928	92.7 (89.3-95.2)	98.4 (98.0-98.7)	81.4 (77.0-85.2)	99.4 (99.2-99.6)	98.0 (97.5-98.4)
Green	221	13	48	3928	82.2 (77.1-86.3)	99.7 (99.4-99.8)	94.4 (90.7-96.7)	98.8 (98.4-99.1)	98.6 (98.1-98.9)

a TP: true positive.

b FP: false positive.

c FN: false negative.

d TN: true negative.

e PPV: positive predictive value.

f NPV: negative predictive value.

### Primary Outcome: High-Risk Red Triage Categorization Performance

For the primary lesion-level analysis of high-risk lesions corresponding to the red triage category, the AI system flagged 68 lesions during screening. Following expert consensus adjudication, 17.6% (n=12) of these lesions were confirmed as true high-risk lesions. In addition, 4 lesions not initially classified as red by the AI system, including 2 lesions not detected by the model and 2 lesions classified as yellow or green, were adjudicated as high risk, yielding 16 high-risk lesions for the primary red triage category analysis.

In the final adjudicated analysis, sensitivity for high-risk lesion identification was 75.0% (95% CI 50.5%-89.8%), and PPV was 17.6% (95% CI 10.4%-28.4%). Specificity, NPV, and overall accuracy were 98.6% (95% CI 98.2%-98.9%), 99.9% (95% CI 99.7%-100%), and 98.5% (95% CI 98.1%-98.8%), respectively (Table 2). In operational terms, the red triage category generated 68 flags among the 4283 interpretable images, corresponding to 15.9 red flags, 2.8 true-positive red flags, and 13.1 false-positive red flags per 1000 interpretable images.

### Secondary Outcomes: Yellow and Green Triage Categorization Performance

In secondary lesion-level analyses based on the final adjudicated reference standard, category-specific performance was evaluated for the yellow and green triage categories. For the yellow category, sensitivity was 92.7% (95% CI 89.3%-95.2%), and PPV was 81.4% (95% CI 77.0%-85.2%); specificity, NPV, and overall accuracy were 98.4% (95% CI 98.0%-98.7%), 99.4% (95% CI 99.2%-99.6%), and 98.0% (95% CI 97.5%-98.4%), respectively. For the green category, sensitivity was 82.2% (95% CI 77.1%-86.3%), and PPV was 94.4% (95% CI 90.7%-96.7%); specificity, NPV, and overall accuracy were 99.7% (95% CI 99.4%-99.8%), 98.8% (95% CI 98.4%-99.1%), and 98.6% (95% CI 98.1%-98.9%), respectively (Table 2).

### Discriminative Performance Based on Detection Confidence

To evaluate discriminative performance across lesion risk categories, ROC curves were constructed using AI detection confidence scores. The expert-adjudicated classification served as the operational clinical reference standard for ROC analysis. For each triage category, a one vs the rest analysis was performed, with the target category treated as positive and all other categories combined as negative. ROC-AUC values were 0.957 for red, 0.931 for yellow, and 0.964 for green (Figure 3). Precision-recall analysis yielded PR-AUC values of 0.8235 for red, 0.9052 for yellow, and 0.9476 for green. The corresponding precision-recall curves are shown in Figure S3 in Multimedia Appendix 1.

**Figure 3.**
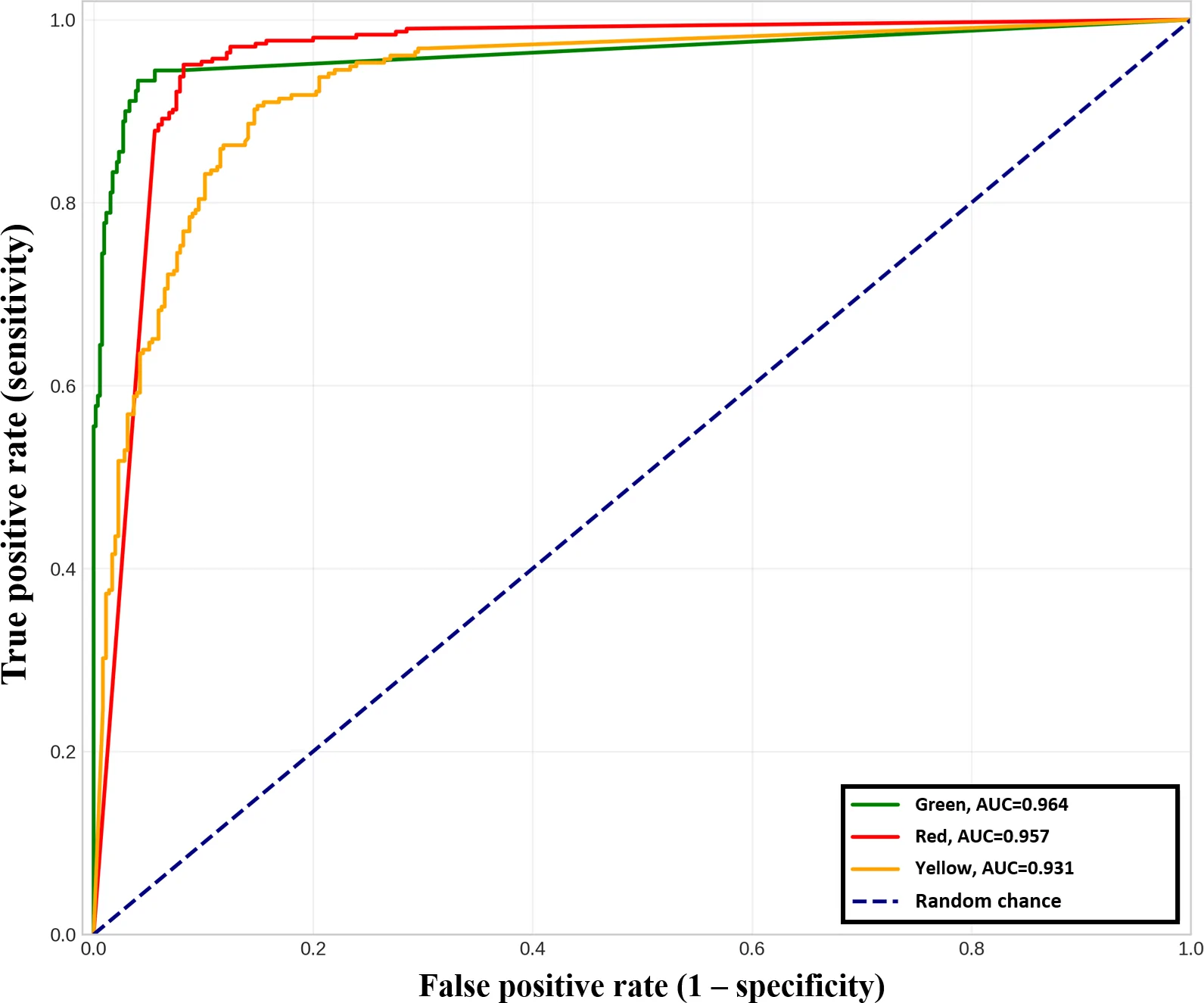
Receiver operating characteristic curves for AI-based oral lesion risk stratification showing the discriminative performance of the AI system for green, yellow, and red risk categories using the expert-adjudicated operational clinical reference standard. The diagonal dashed line represents random classification. AUC: area under the curve.

## Discussion

### Principal Findings

In this prospective community-based field evaluation study, we assessed a previously developed, parameter-locked, mobile AI-assisted oral lesion triage system within a routine community OC screening workflow. Under field conditions, including image acquisition by trained nonspecialist personnel using handheld mobile devices, on-site image quality assurance, AI-assisted lesion-level risk stratification, and expert adjudication, the system was feasibly integrated into the screening workflow and provided preliminary lesion-level performance estimates for the high-risk red category. The primary red triage category analysis was based on 16 high-risk lesions and showed moderate sensitivity with modest PPV, reflecting the low-prevalence structure of community screening. Unlike model development or retrospective validation studies, this study evaluated how an AI-assisted triage system functioned when embedded in a real-world mobile screening workflow, where image quality, operator experience, lesion distribution, and expert interpretation reflect frontline community practice. These findings suggest that mobile AI-assisted oral lesion triage may help bridge the gap between algorithmic validation and practical field evaluation. The system should therefore be understood not as an autonomous diagnostic tool but as human-supervised triage support within a digital health workflow designed to assist risk stratification and referral prioritization while preserving expert oversight.

### Interpretation in the Context of Community Screening

Community-based OC screening differs fundamentally from the controlled image datasets commonly used in AI research. Prospective field evaluation can therefore reveal deployment-related constraints, including acquisition variability, low-prevalence predictive values, and reference standard uncertainty, that may not be fully captured in model development or retrospective validation studies [29,30]. More broadly, recent clinical AI evaluation commentaries have similarly emphasized that aggregate metrics or direct output-only evaluation may be insufficient to assess workflow-level reliability, interpretability, and deployment safety [31,32]. In routine mobile screening workflows, intraoral images are acquired on-site by trained nonspecialist personnel under variable lighting, positioning, oral opening, and operator conditions. In addition, the prevalence of high-risk lesions within community screening populations is low, resulting in a predominance of normal or nonspecific findings and a markedly imbalanced risk distribution. In this study, despite high overall preconsensus interrater agreement, disagreement among the 3 independent specialists was observed for 112 lesions before consensus adjudication, underscoring that a subset of oral mucosal findings remained clinically equivocal even under expert review. The contrast between the preconsensus summary and the final adjudicated analysis illustrates the clinical uncertainty inherent in field screening and supports the need for structured expert adjudication when evaluating AI-assisted triage systems. Within such workflows, image quality assurance is a central deployment requirement because reliable risk stratification depends not only on model performance but also on the quality and consistency of field-acquired images.

### Interpretation of Predictive Values in Low-Prevalence Screening

The high-risk red triage category showed moderate sensitivity and modest PPV despite high specificity and NPV. This finding should be interpreted within the epidemiologic structure of community-based OC screening. Population-based studies from Taiwan have reported that the prevalence of highly suspicious or referral-requiring oral lesions in community screening settings is approximately 0.9% to 2.2% [26-28]. In such low-prevalence settings, even screening tools with high sensitivity and specificity may yield constrained PPVs because predictive values are strongly influenced by baseline disease prevalence [33,34]. Therefore, the PPV observed for the red triage category should not be interpreted as a stand-alone diagnostic measure. It should also not be interpreted as an operational measure of positive triage yield and expert review burden. Rather, it should be considered in relation to the intended role of AI-assisted triage: prioritizing a smaller subset of lesions for further specialist evaluation while preserving human oversight in a predominantly low-risk screening population [35-37].

### Contextual Risk Enrichment and Quality Management Relevance

As an exploratory contextual analysis, PPV lift was used to describe the degree of risk enrichment achieved by the red triage category relative to the expected baseline event rate in the screened population. Using reported baseline prevalence estimates for high-risk oral lesions in Taiwanese community screening populations and the observed red category sensitivity and specificity, the estimated PPV lift suggested that AI-assisted triage may concentrate high-risk findings within the red category [38-40]. PPV has also been used as a quality management indicator in Taiwan’s oral mucosal screening program, although such benchmarks were developed for program-level screening evaluation rather than lesion-level AI triage assessment [41,42]. Although direct comparison is limited by differences in outcome definitions, analysis level, and reference standards, the lesion-level PPV of 17.6% observed for AI red triage may be viewed as a contextual signal of risk enrichment under low-prevalence community screening conditions. Because the present analysis did not directly measure participant-level referral yield, specialist workload, cost-effectiveness, or downstream clinical outcomes, this finding should be interpreted as a potential prioritization signal within a supervised triage workflow rather than direct evidence of clinical benefit.

### Human-Supervised Triage and Expert Oversight

The structured expert adjudication process also provides insights into how AI outputs were incorporated into the screening workflow. Prior studies have cautioned that AI-driven decision support may introduce automation bias or anchoring effects if clinicians overrely on model outputs, particularly when explainability alone is insufficient to ensure appropriate clinical interpretation [43]. In the present study, the presence of 112 discordant lesions before consensus adjudication and the identification of 4 AI-missed high-risk lesions during expert review suggest that expert review included active assessment beyond AI-flagged outputs; however, because reviewers were aware of AI outputs, the process remains susceptible to AI-related review or incorporation bias. Instead, discordant or equivocal findings were subjected to formal consensus adjudication, and additional high-risk lesions not classified as red by the AI system were incorporated into the final operational clinical reference standard. This process helped maintain a practical distinction between AI-generated triage suggestions and expert-derived clinical reference decisions within the workflow. These findings are consistent with broader recommendations that medical AI systems should support rather than replace clinical judgment, with appropriate oversight and accountability maintained throughout deployment [44-46].

### Failure Patterns and Field Deployment Implications

The 4 lesions adjudicated as high risk but not initially classified as red by the AI system provide clinically informative examples of deployment-relevant limitations under mobile field screening conditions. These cases included lesions with borderline visual characteristics, such as mixed erythematous and leukoplakic appearances, lesions located near image margins, and lesions with limited local contrast or incomplete visibility. These findings suggest that subtle morphology, partial lesion capture, peripheral lesion location, and low contrast may reduce model sensitivity for high-risk triage in field-acquired images. From a workflow perspective, these failure patterns underscore the importance of maintaining human oversight, comprehensive image review, and structured expert adjudication in AI-assisted community screening, particularly for lesions that fall along a continuum of clinical risk. Representative examples are shown in Figure 4.

**Figure 4.**
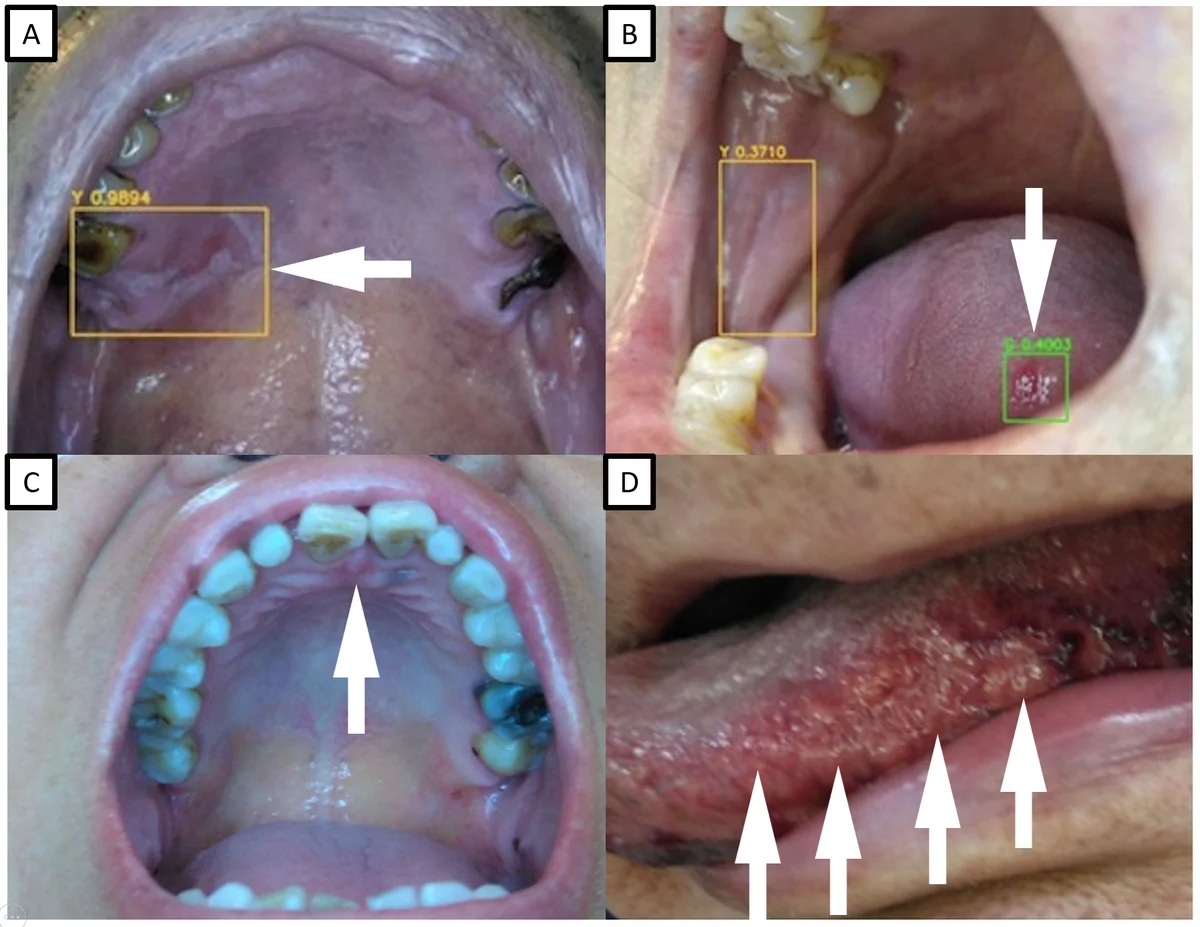
Representative false-negative cases in the high-risk (red) triage category during AI-based oral cancer screening (n=4): (A) a borderline lesion with mixed erythematous and leukoplakic appearance that was initially classified by the AI system as low risk (yellow) and subsequently reclassified as high risk (red) following expert consensus; (B) a lesion (green) located near the image margin that was not flagged as high risk by the AI system, where proximity to the image boundary may have influenced model detection; and (C and D) lesions not identified by the AI system that were subsequently recognized by experts upon case-by-case image review at the locations indicated by the white arrows and classified as high risk.

### Practical Implications for Community Screening

The findings of this study support the potential value of AI-assisted oral lesion triage in remote community screening settings, where access to specialist oral oncology expertise may be limited. By combining on-site image quality assurance, risk-stratified AI outputs, and expert adjudication, this mobile screening workflow may support referral prioritization under specialist oversight rather than directly establishing improved diagnostic performance by frontline personnel. Because the fixed red triage operating point yielded modest PPV and measurable false positive review burden, future implementation studies should evaluate whether alternative operating points could better balance sensitivity, PPV, and specialist workload. Within Taiwan’s existing national OC screening program, such systems could serve as adjunctive tools for referral prioritization rather than replacements for clinical judgment. This approach may be particularly relevant for high-risk or geographically underserved populations, where scalable triage support could help bridge gaps between community screening and specialist evaluation. Further studies are needed to determine whether mobile AI-assisted triage can improve referral efficiency, specialist workload, cost-effectiveness, and downstream clinical outcomes.

### Limitations

Several limitations warrant consideration. First, the primary performance analysis was conducted at the lesion level rather than as a participant-level clinical outcome analysis. Although this approach was aligned with the lesion-level AI outputs and expert adjudication used in the present workflow, future studies should evaluate participant-level referral decisions, referral completion, and downstream clinical outcomes. Second, histopathologic confirmation was not systematically obtained for all lesions or screened participants. Expert consensus therefore served as an operational clinical reference standard for triage evaluation rather than a definitive diagnostic gold standard. Third, expert adjudication was not blinded to AI outputs, reflecting the workflow-integrated design of this study but introducing potential AI-related review or incorporation bias. This unblinded operational reference standard may have influenced expert classification and may have overestimated agreement between AI triage and expert adjudication. Fourth, the number of high-risk lesions was limited, with only 16 high-risk lesions in the primary red triage category analysis. This was consistent with the low-prevalence structure of community screening but constrained the precision of sensitivity and PPV estimates. Fifth, performance analysis was restricted to interpretable images. Images that remained technically inadequate after repeated acquisition attempts were excluded, which may have introduced selection bias and may underestimate the practical burden of image acquisition in field screening. Sixth, this was a single-region field evaluation, and mobile device characteristics, operator training, acquisition conditions, local screening infrastructure, and population risk profiles may affect generalizability to other settings. Individual-level demographic variables and behavioral risk factors were not collected in the analytic dataset, precluding subgroup analyses by age, sex, tobacco use, alcohol use, or betel quid use. Finally, this study did not include a usual care comparator arm; direct evaluation of nonspecialist diagnostic performance; or formal measurement of acquisition time or repeat acquisition rate, specialist workload, cost-effectiveness, or longitudinal outcome linkage. Future multicenter studies incorporating blinded assessment, workflow efficiency metrics, registry linkage, and longitudinal follow-up will be needed to evaluate generalizability, clinical impact, and scalability.

### Conclusions

In this prospective community-based field evaluation, a parameter-locked mobile AI-assisted oral lesion triage system was integrated into a routine OC screening workflow operated by trained nonspecialist personnel. Under field conditions characterized by mobile image acquisition, low prevalence of high-risk lesions, and variability in image quality and lesion presentation, the system provided preliminary lesion-level evidence of red triage categorization performance, with moderate sensitivity and modest PPV for high-risk lesion identification. These findings suggest that mobile AI-assisted triage may support referral prioritization within a human-supervised community screening workflow while preserving the need for expert oversight. Further multicenter studies with blinded assessment, participant-level outcome evaluation, workflow efficiency measures, and longitudinal follow-up are needed to determine the clinical impact and scalability of AI-assisted OC screening in real-world practice.

## Supplementary material

10.2196/101721 Multimedia Appendix 1 Supplementary methods, model training outcomes, representative community screening environment, representative AI-generated triage outputs, and precision-recall curves.

10.2196/101721 Checklist 1 STARD 2015 checklist.

## Abbreviations

NPV: negative predictive value
OC: oral cancer
OPMD: oral potentially malignant disorder
PPV: positive predictive value
PR-AUC: area under the precision-recall curve
ROC: receiver operating characteristic
ROC-AUC: area under the receiver operating characteristic curve
STARD: Standards for Reporting of Diagnostic Accuracy Studies
