# Supplementary material for: Mobile AI-Assisted Oral Lesion Triage for Community Oral Cancer Screening: Prospective Field Evaluation Study
Source: JMIR Mhealth Uhealth. 2026 Oct 20;14:e101721. doi: 10.2196/101721 (PMC13683989; doi:10.2196/101721)
Supplement: Supplementary file 1 [file mhealth-v14-e101721-s001.docx]

**Supplementary Methods**

**Training strategies**

During DINO model training, a contrastive denoising training strategy was used. Random noise was added to the ground-truth bounding boxes during training, and contrastive learning was used to guide the model in recovering the original annotations. This strategy was designed to improve robustness to annotation noise and enhance detection of small objects. In addition, DINO was trained with a Mixed Query Selection algorithm, which incorporates both spatial and semantic information to generate diverse query vectors and improve attention to relevant image regions.

All models were trained using the same hyperparameter settings, including learning rate, batch size, and number of training epochs. The AdamW optimizer was used throughout. Model training and inference were conducted on a workstation equipped with an NVIDIA RTX 2080 GPU with 12 GB VRAM, an Intel Xeon Silver 4110 CPU, and 93 GB RAM. The training outcomes are shown in Table S1.

Table S1. Training outcomes of previous and current oral lesion triage models.

| Model | Accuracy | | | mAP@0.5 |
| --- | --- | --- | --- | --- |
|  | Red | Yellow | Green |  |
| YOLOv7-d6-EMA | 93% | 84% | 89% | 0.747 |
| DINO | 95% | 80% | 78% | 0.777 |
| DINO-YOLO MoE | 95% | 83% | 84% | 0.798 |
| YOLOv7 DINO MoE (current model) | 98% | 95% | 99% | 0.805 |

**Figure S1.** Real-world community-based oral cancer screening environment in a remote setting.

**Figure S2.** Representative AI-generated triage outputs for green, yellow, and red categories. (A) Green category (torus palatinus), indicating no actionable lesion or no evident malignant potential. (B) Yellow category (homogeneous leukoplakia), indicating a lesion requiring structured follow-up. (C) Red category (oral erythroplakia), indicating a lesion requiring specialist referral because of high-risk clinical features. Bounding boxes indicate regions identified by the AI system. The examples are intended to show how the triage outputs appear in practice and are not meant to cover all possible oral mucosal findings.

Figure S3. Precision-recall curves for AI-based oral lesion risk stratification across red, yellow, and green triage categories, using the expert-adjudicated operational clinical reference standard. Each category was evaluated using a one-versus-rest framework.
